# Hypoxia destroys the microstructure of microtubules and causes dysfunction of endothelial cells via the PI3K/Stathmin1 pathway

**DOI:** 10.1186/s13578-019-0283-1

**Published:** 2019-02-18

**Authors:** Huaming Cao, Dongsheng Yu, Xueyun Yan, Bing Wang, Zhiming Yu, Yu Song, Liang Sheng

**Affiliations:** 1 Department of Cardiology, Shibei Hospital, 4500 Gong He Xin Road, Shanghai, 200435 China; 2 Department of Traditional Chinese Medicine, The First Affiliated Hospital of Zhengzhou University, Zhengzhou, 450052 Henan China; 3 Department of Hepatobiliary and Pancreatic Surgery, The First Affiliated Hospital of Zhengzhou University, Zhengzhou, 450052 China; 4 Henan Key Laboratory of Digestive Organ Transplantation, Zhengzhou, 450052 China; 5 Department of Cardiology, Wuxi People’s Hospital Affiliated to Nanjing Medical University, Wuxi, 214023 Jiangsu China; 6 Pharmacy College, Xinxiang Medical University, 601 Jinsui Avenue, Hongqi District, Xinxiang, 453000 Henan China

**Keywords:** HUVEC, Hypoxia, Microtubule, PI3K, Stathmin1

## Abstract

**Background:**

Endothelial cells (EC) are sensitive to changes in the microenvironment, including hypoxia and ischemia. Disruption of the microtubular network has been reported in cases of ischemia. However, the signaling pathways involved in hypoxia-induced microtubular disruption are unknown. The purpose of this study was to investigate the molecular mechanisms involved in hypoxia-induced microtubular disassembly in human umbilical vein endothelial cells (HUVECs).

**Results:**

HUVECs were cultured under normoxic or hypoxic conditions and pretreated with or without colchicine or paclitaxel. The MTT assay, Transwell assay, trans-endothelial permeability assay, and 5-bromo-2′-deoxy-uridine staining were used to test the survival rate, migration, permeability, and proliferation of cells, respectively. Transmission electron microscopy and phalloidin staining were used to observe the microstructure and polymerization of microtubules. The results show that the functions of HUVECs and the microtubular structure were destroyed by hypoxia, but were protected by paclitaxel and a reactive oxygen species (ROS) inhibitor. We further used western blot, a luciferase assay, and co-immunoprecipitation to describe a non-transcription-independent mechanism for PI3K activation-inhibited microtubular stability mediated by Stathmin1, a PI3K interactor that functions in microtubule depolymerization. Finally, we determined that hypoxia and ROS blocked the interaction between PI3K and Stathmin1 to activate disassembly of microtubules.

**Conclusion:**

Thus, our data demonstrate that hypoxia induced the production of ROS and damaged EC function by destroying the microtubular structure through the PI3K/stathmin1 pathway.

## Background

Blood vessels play a major role maintaining oxygen and nutrient supply to all tissues in the body. The endothelium is a single layer of endothelial cells (ECs) that lines the blood vessel lumen and controls vessel function. Vessel hypoxia emerges when changes in supply or demand occur during normal mammalian development and human diseases [1]. Hypoxia and hypoxia-inducible factor (HIF) signaling regulate multiple aspects of EC biology, including cell survival, growth, invasion, glucose metabolism, vascular tone, and barrier, which contribute to the induction of angiogenesis [2].

The inability of ECs to perform their physiological functions (EC dysfunction) contributes to cardiovascular disease [3]. ECs are sensitive to changes in the microenvironment (e.g., ischemia and hypoxia), which is usually paralleled with oxidative stress. Oxidative stress resulting from activation of cellular reactive oxygen species (ROS) production is a part of the pathological course of hypoxia. Uncontrolled ROS production causes tissue damage, vascular barrier dysfunction, and inflammation [4–6].

Microtubule dynamics also control fundamental cellular functions, such as cell shape, polarity, motility, migration and division, as well as participate in other aspects of EC biology [7]. The integrity of the microtubular system is necessary for protein trafficking, further affecting the frequency and velocity of vesicle transport and altering plasma membrane composition through their direct effects on membrane trafficking pathways. The EC microtubular network plays a role in vascular permeability. Disruption of microtubules with nocodazole promotes barrier dysfunction, which is attenuated by pretreatment with forskolin [8]. Furthermore, tumor necrosis factor (TNF)-, thrombin-, and transforming growth factor (TGF)-induced endothelial permeability is associated with destabilization of tubulin and the peripheral microtubular network [9].

Stathmin1 is a cytosolic 19-kDa phosphoprotein that plays an oncogenic role and acts as a prognostic marker in several kinds of cancers. Stathmin1 is overexpressed in tumor tissues and is correlated with cancer progression and poor prognosis by regulating cell division, motility, and migration, all of which are critical processes in ECs [7, 10]. Stathmin1 is a ubiquitous cytoplasmic phosphoprotein that regulates microtubular dynamics by the depolymerization effect [11] and is a significant marker of activation of the phosphatidylinositol 3-kinase-Akt pathway (PI3K-Akt) pathway [12]. The PI3K/Akt pathway has been linked to an extraordinarily diverse group of cellular functions, including cell growth, proliferation, differentiation, motility, survival, and intracellular trafficking [13]. Mammalian class I PI3K can be divided into classes IA and IB; the class IA PI3Ks are heterodimers of a 110 kDa catalytic subunit (p110α, p110β, or p110δ) and a regulatory subunit of 85 or 55 kDa (p85/p55) [13]. P110α PI3K (PIK3CA) has an important role in EC cell migration and angiogenesis [14], so the dominant form of class IA PI3K was used in our study to enhance PI3K activity and observe the effect on microtubular stability.

Previous studies focused on the effect of hypoxia, reactive oxygens species (ROS) or microtubular disassembly on proliferation, survival, and permeability of ECs [15, 16], but very few studies have connected all of these factors together to explore the internal interaction. In this study, we utilized an in vitro simulated hypoxic model in HUVECs [17], along with a microtubule inhibitor and stabilizer, to investigate the association between the generation of ROS and microtubular dynamics. We focused on Stathmin1 to investigate its functional role and the molecular mechanism involved in EC dysfunction.

## Materials and methods

### Cell culture

HUVEC were obtained from ATCC (Manassas, USA) and cultured in Medium 199 (Invitrogen, Carlsbad, CA, USA) containing 10% (v/v) fetal bovine serum (FBS, HyClone, UT, USA) at 37 °C. For hypoxia experiments, cells were subjected to Earle’s solution (NaCl 116.4 mM, KCl 5.4 mM, CaCl_2_ 1.8 mM, MgSO_4_ 0.8 mM, NaH_2_PO_4_ 2.6 mM, NaHCO_3_ 26.2 mM, Hepes 20.1 mM, pH7.4) [18] saturated by 95% N_2_ plus 5% CO_2_ for 6 h in a Tri-gas hypoxia incubator (Forma Scientific, Marietta, OH, USA). ROS inhibitor, N-acetyl cysteine (NAC, #A0737), hydrogen peroxide solution (#323381) and cycloheximide (#5087390001) was purchased from Sigma-Aldrich (Steinheim, Germany). Paclitaxel (#S1150), colchicine (#S2284) and wortmannin (#S2758) were obtained from Selleck (Shanghai, China), Actinomycin D (#A4448) was purchased from Apexbio (Shanghai, China). PIK3CA-WT, PIK3CA-H1047R and E545K mutation were gifts from Joan Brugge (Addgene plasmid # 14572) [19]. PI3K siRNA, stathmin1 siRNA and PIK3CA siRNA were designed and synthesized from GenePharm Co, Ltd. (Shanghai, China).

### Transwell assay

HUVEC migration was evaluated with transwell system (Corning Costar, MA, USA). Briefly, 1 × 10^5^ HUVECs were seeded in the upper chambers for 12 h attachment. Then cells were subjected to hypoxic condition and chemical treatment for 6 h. The medium in upper chambers was switch to M199 with 0.5% FBS, while, that in the lower chamber were switch to M199 with 1% FBS. After 12 h incubation, the cells on the bottom were fixed with 4% paraformaldehyde and stained with 1% crystal violet, the non-migrating cells in the upper chamber were removed. Finally, the crystal violet was dissolved in 33% acetic acid, and the absorbance was measured at 600 nm. The amount of cell migration was determined as the ratio of the OD values of the treatment relative to the control. Each treatment was repeated in three independent chambers.

### Trans-endothelial permeability assay

Cells were grown on 0.2 mm pore-size collagen IV (1 mg/mL)-coated tissue culture inserts (Nunc, Fisher Scientific, Pittsburgh, PA) until confluent. Monolayers were then serum-starved for 1 h and either left untreated or exposed to hypoxic condition in triplicate with desired agents. Following treatment, fluorescein isothiocyanate (FITC) dextran (10 kDa) dissolved in the medium was placed in the upper chamber at a concentration of 0.4 mg/mL and allowed to equilibrate for 2 h. Samples were then taken from the lower chamber for fluorescence measurements. Fluorescence was measured by excitation at 492 nm and the emission collected at 520 nm.

### Transmission electron microscopy (TEM)

After treated, HUVECs were fixed for 1 h with cold 3% glutaraldehyde in 0.1 M cacodylate buffer (pH 7.3) and washes with 0.2 M cacodylate, and then cells were pelleted and then postfixed with 2% osmium tetroxide in 0.1 M cacodylate for 1 h at 4 °C, stained en bloc with 2% uranyl acetate for an additional 1 h. After three more washes in double-distilled water, the samples were dehydrated in a series of acetone solutions and embedded in Epon 812 according to the standard procedure. Ultrathin sections (70 nm) were prepared, stained with both uranyl acetate and lead citrate, and assessed using a Hitachi 7400 electron microscope (Hitachi, Tokyo, Japan). Random fields taken from individual samples were photographed at 30,000×.

### ROS assay

After hypoxia treatment, 5 μM dichlorofluorescein diacetate (DCF) was added into HUVEC cells culture medium and incubate for 1 h at 37 °C. Then cells were washed cells twice with PBS and scrape cells into RIPA buffer and incubate for 10 min on ice. After spin at 13,000 rpm for 10 min, 100 μl supernatant was transferred into 96-well plates for fluorescence assay (485 nm excitation and 527 nm emission). Protein concentration was tested for the normalization of cell number.

### 5-Bromo-2′-deoxy-uridine (BrdU) incorporation assays

HUVEC cells were subjected to hypoxic condition and chemical treatment for 6 h and then incubated with 0.1 mg/ml BrdU for 3 h before fixed by 4% paraformaldehyde (PFA). Cells were incubated in 0.5% Triton X-100, 1.5 N HCl, and washed by PBS between incubation, then add 0.25% trypsin EDTA, and incubate at 37 °C for 5 min. Cells were washed by PBS and blocked in blocking buffer for 2 h. BrdU antibody (1:100, ab6326, Abcam) was used and then incubated overnight at 4 °C in a hydration chamber. After wash with PBST, secondary antibody containing 2-(4-amidinophenyl)-6-indolecarbamidine dihydrochloride (DAPI) were added and incubated at RT for another 3 h, then observed with Olympus IX81.

### Phalloidin staining

HUVEC cells were plated on fibronectin (10 µg/mL)-coated glass coverslips treated with indicated condition. Attached cells were fixed with 4%PFA, followed by permeabilization with 0.2% Triton X-100 at room temperature for 10 min, cells were stained with 100 nM Alexa Fluor™ 488-conjugated phalloidin (A12379, Thermo Fisher) in dark for 30 min and then incubated with DAPI at room temperature for 10 min. After wash with PBS, stained cells were examined by confocal microscope.

### MTT assay for cell proliferation

The 3-(4,5-dimethylthiazol-2-yl)-2,5-diphenyltetrazolium bromide (MTT) assay was used to determine the effect of treatment on cell viability. HUVEC were inoculated into 96-well plates at 5 × 10^4^ cells/ml, after treated with hypoxia and chemicals, 5 mg/ml MTT (Beyotime Biotech, Jiangsu, China) was added into the cell culture medium. After further 4 h incubation, the supernatant fraction was removed and 150 µl of dimethyl sulphoxide (DMSO) was added. The optical density (OD) at 490 nm was measured using a microplate reader (Bio-Rad, Hercules, CA, USA).

### Western blot analysis

Total lysates from HUVEC cells were obtained by lysing in RIPA buffer with protease inhibitors cocktail (#HY-K0010, MedChem Express, Shanghai, China). Protein concentration was measured by the BCA assay (Bio-Rad, Hercules, CA, USA). Proteins were extracted and separated in 10% Tris glycine/SDS–polyacrylamide gels and electro-transferred to ECL nitrocellulose membranes (#IPFL00010, Millipore, Bedford, MA, USA). The membranes were blocked with 5% nonfat milk and incubated with specific antibodies overnight at 4 °C. β-Actin or P85 was used as the endogenous control. Primary antibodies were used at the dilution of 1:1000. Anti-Phospho-Akt (Ser473) (#4060), Akt (#4685), PI3K (#4249), stathmin1 (#13655) were purchased from Cell Signaling Technology (Beverly, MA, USA). Anti-β-actin (ab8266), anti-P85 (ab191606), anti-α Tubulin (acetyl K40) (ab24610) and horseradish peroxidase-conjugated anti-mouse or rabbit IgG were purchased from Abcam (Cambridge, MA, USA).

### RT-qPCR

Total RNA was extracted from cells using RNAiso Plus (Takara Bio Inc., Dalian, China) and was reverse-transcribed using M-MMLV Reverse Transcriptase (#A3500, Promega Biotech Co., Ltd, Beijing, China) according to the manufacturer’s protocols. RT-qPCR was performed using SYBR (#4385612, Thermo Scientific Inc.). Primer sequences used in the experiments were as follows: Stathmin 1 (homo) forward 5′-TCAGCCCTCGGTCAAAAGAAT-3′, reverse 5′-TTCTCGTGCTCTCGTTTCTCA-3′; PPIA (homo) forward 5′-CCCACCGTGTTCTTCGACATT-3′, reverse 5′-GGACCCGTATGCTTTAGGATGA-3′. The expression levels of mRNA were normalized to PPIA mRNA.

### Statistic methods

The data are expressed as mean ± SEM. All experiments were performed in triplicate. All statistical analyses were performed with the SPSS 19.0 using non-parametric tests. The Kruskall Wallis test followed by the Mann–Whitney test was used to detect differences between groups. *P *< 0.05 was considered statistically significant.

## Results

### Hypoxia destroys microtubular ultrastructure and induces HUVEC dysfunction

**Fig. 1:**
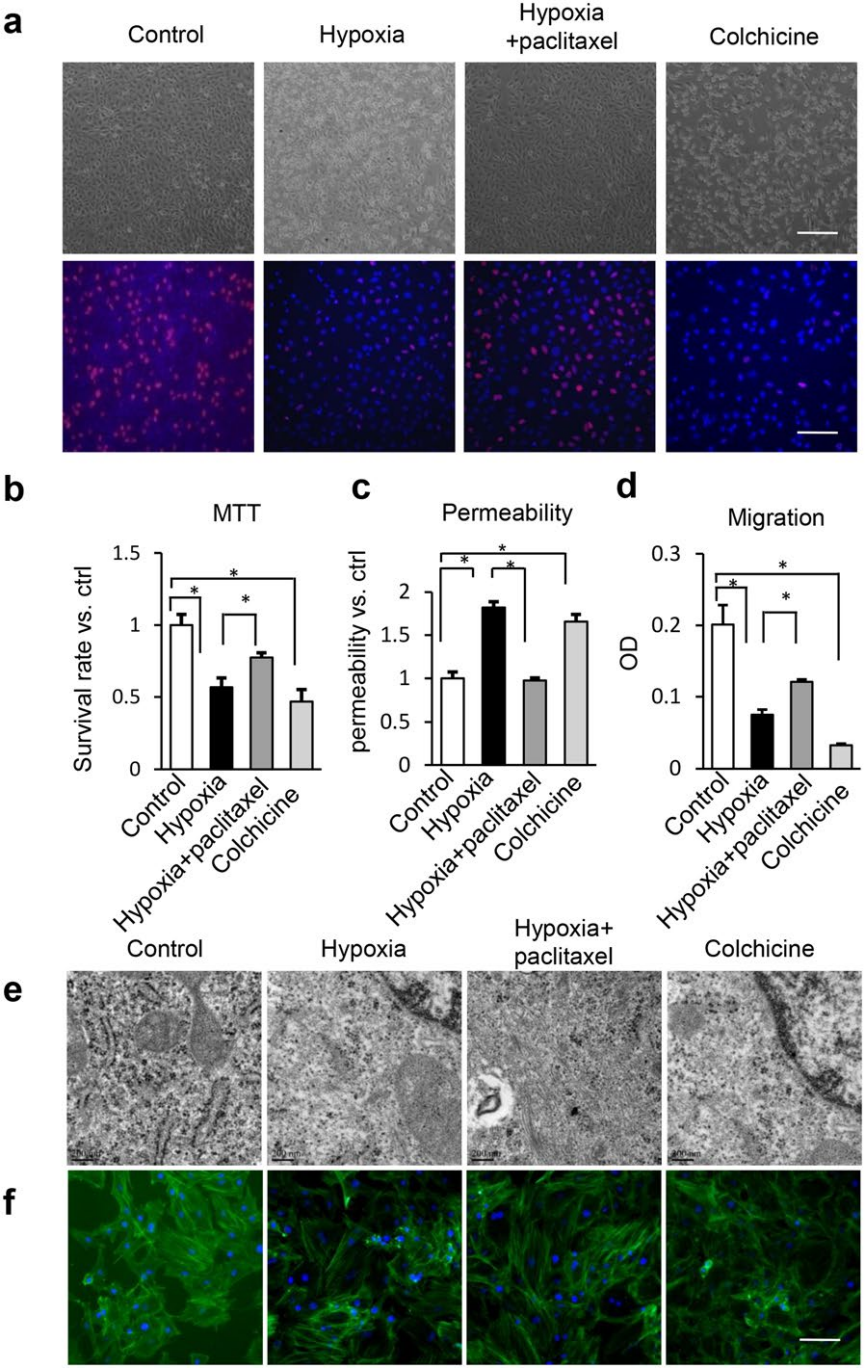
Microtubule stabilization reverses the reduction of proliferation, survival rate, migration, barrier integrity and microtubular depolymerization induced by hypoxia in HUVECs. HUVECs were cultured under normoxic or hypoxic conditions with or without pretreatment of 10 μM paclitaxel or 4 μM colchicine for 1 h. **a** Cells were immunostained by BrdU antibody and DAPI for proliferation assay. Bar = 50 μm. **b** The survival rate (%) of HUVECs was determined using MTT assay. **c** Permeability of HUVECs was tested in cell culture inserts through determining the fluorescence leaked into lower chamber. All the data were normalized to control group. **d** Cell migration was tested by transwell method. **e** HUVECs were processed for transmission electron microscopy (TEM) to examine microtubular structure, bar = 200 nm. **f** Hypoxia-induced microtubule depolymerization was shown by immunofluorescent confocal micrographs in HUVEC cells. Cells were stained with phalloidin (green) and the nuclear stain DAPI (blue). Bar = 50 μm. Representative results from three independent experiments were depicted here. The data are presented as the mean ± SEM. *P < 0.05

Microscopic images revealed the increase in mortality of HUVECs under a hypoxic condition, similar to those treated with colchicine, a reagent that disassembles microtubules. Paclitaxel, a microtubule stabilizer, reversed the mortality of HUVECs under the hypoxic condition (Fig. 1a upper). Proliferation of HUVECs was further confirmed by BrdU staining. Hypoxia and colchicine treatment significantly inhibited the percentage of BrdU-positive cells, but paclitaxel reversed the hypoxia-induced inhibitory effect (Fig. 1a lower). Proliferation rate was further confirmed by the MTT assay (Fig. 1b). Migration and barrier integrity, important functional indices of venous ECs during vascular growth and repair, were also damaged during the hypoxia and colchicine treatments, while paclitaxel reversed the malfunction induced by hypoxia (Fig. 1c, d). Furthermore, microtubular structures were observed by transmission electron microscopy (TEM) and phalloidin staining and the results showed that hypoxia destroyed microtubular ultrastructure and destabilized microtubule polymerization (Fig. 1e, f). These results suggest that hypoxia inhibits proliferation and induces malfunction of HUVECs through disassembly of microtubules.

### Hypoxia induces ROS production causing HUVEC malfunction

**Fig. 2:**
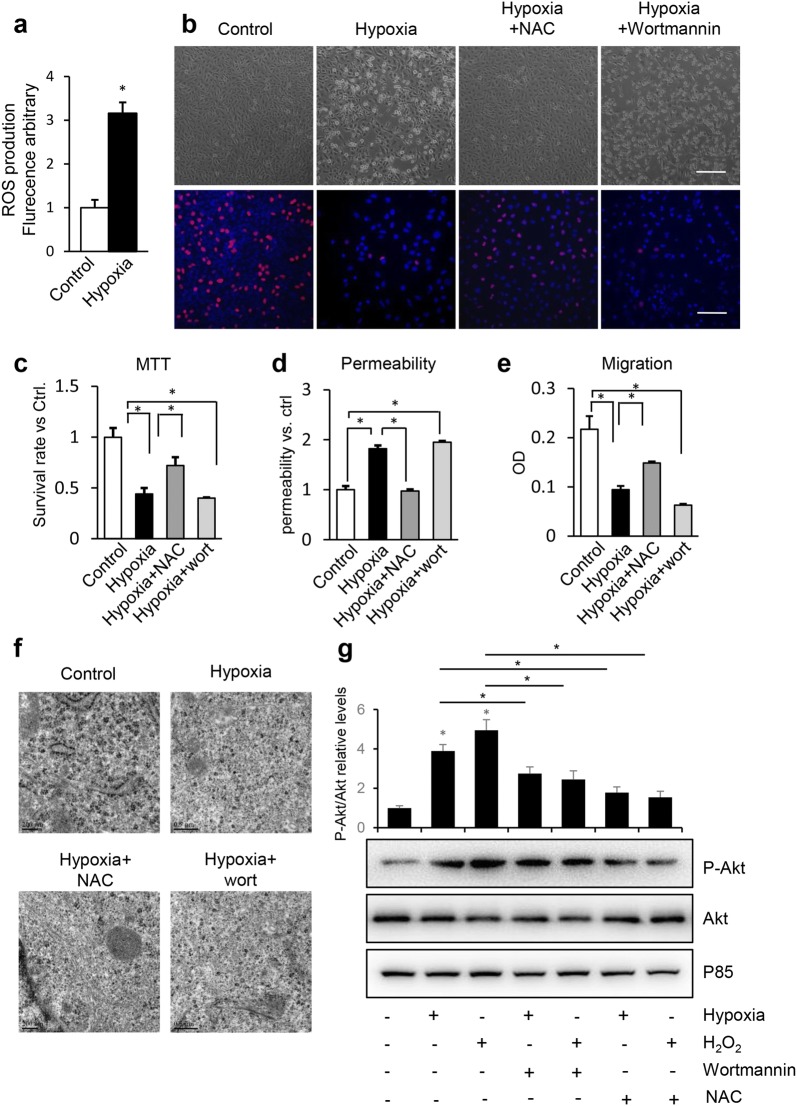
Hypoxia induces the production of ROS, which reduces the proliferation, survival rate, migration, barrier integrity and microtubular depolymerization in HUVECs. HUVECs were cultured under normoxia or hypoxia condition with or without pretreatment of *N*-acetyl cysteine (NAC, 1 mM) or wortmannin (1 μM) for 30 min. **a** ROS production in HUVECs. **b** Microscopic observation and BrdU staining assessed the HUVECs proliferation. Bar = 50 μm. **c** The survival rate (%) of HUVECs determined using the MTT assay. **d** Permeability of HUVECs was tested in cell culture inserts through determining the fluorescence leaked into lower chamber. All the data were normalized to control group. **e** Cell migration was tested by transwell method. **f** HUVECs were processed for TEM to examine microtubule structure. Bar = 0.5 μm. **g** HUVECs were cultured under normoxia, hypoxia or H_2_O_2_ conditions with or without pretreatment of 1 mM NAC or 1 μM wortmannin for 30 min, total cell lysates were immunoblotted with antibodies to P-Akt, Akt and P85. Representative results from three independent experiments are depicted here. The relative intensity of band was normalized to P85. The data were presented as the mean ± SEM. *P < 0.05

Hypoxia damages mitochondrial function, leading to leakage of electrons from the respiratory chain, which increases ROS. Thus, the ROS level increased remarkably in HUVECs under the hypoxic condition (Fig. 2a), and the survival rate of HUVECs increased in response to N-acetylcysteine (NAC) and wortmannin treatment according to the microscopic observations and the MTT assay (Fig. 2b, c). The NAC and wortmannin treatments also increased proliferation and migration, but reduced the permeability of HUVECs under the hypoxic condition (Fig. 2d, e). Hypoxia induced microtubular disassembly in HUVECs through ROS, whereas NAC and wortmannin protected against destruction of the microtubular ultrastructure induced by hypoxia (Fig. 2f). A previous study showed that ROS activates the PI3K/Akt pathway in HUVECs [20], so we were interested in determining whether hypoxia and H_2_O_2_ treatment could also activate the PI3K/Akt pathway in HUVECs. As shown in Fig. 2g, both hypoxia and H_2_O_2_ increased phosphorylation of Akt, and 1 mM NAC or 1 μM wortmannin for 30 min decreased the expression of P-Akt, but NAC downregulated the phosphorylation rate to a lower level than wortmannin. This result indicates that ROS caused microtubular disassembly under the hypoxic condition, and connected the hypoxia and malfunction of HUVECs through the PI3K/Akt pathway.

### Stathmin1 is downregulated after hypoxia and H_2_O_2_ treatments

**Fig. 3:**
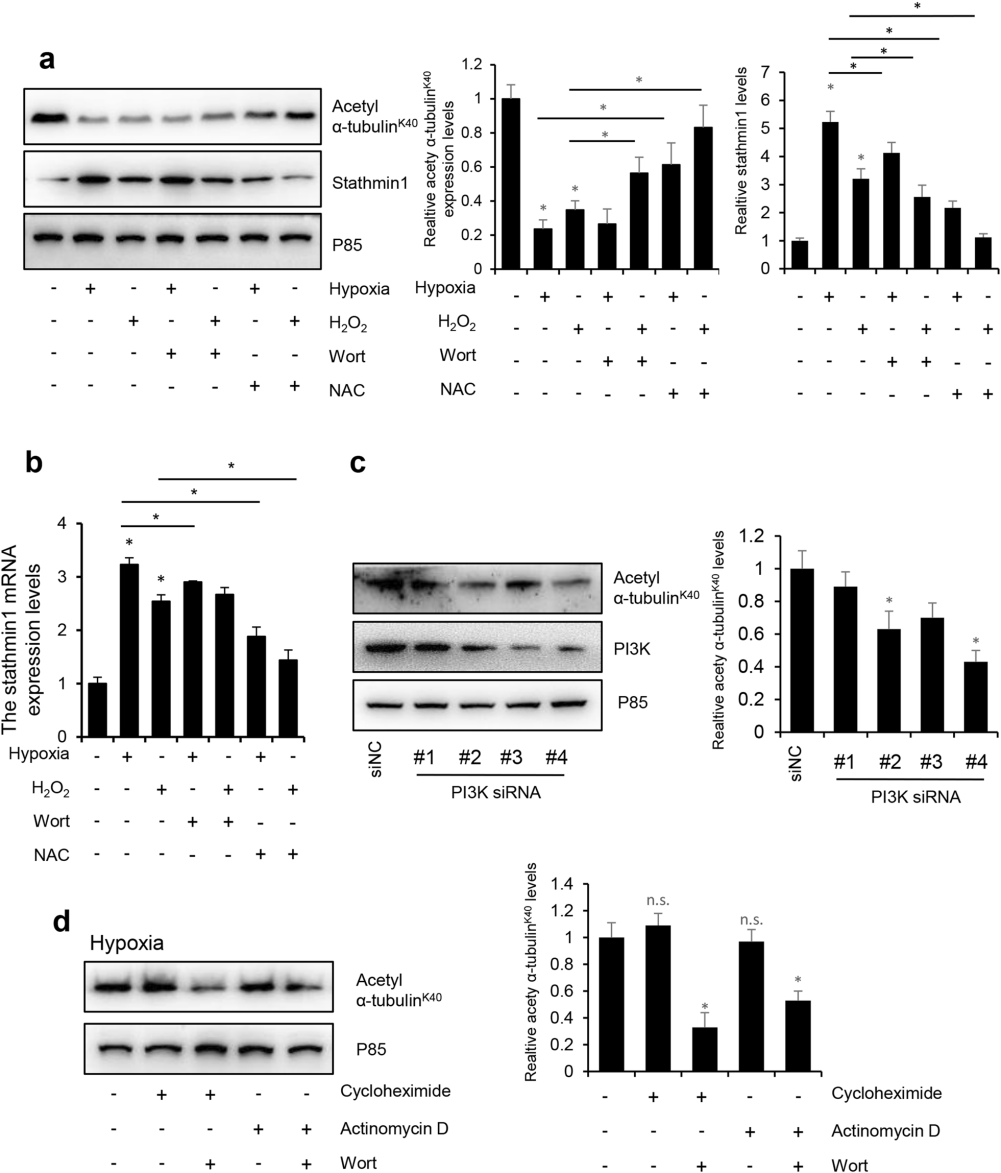
Stathmin1 is down-regulated after hypoxia and H_2_O_2_ treatment. HUVECs were cultured under normoxia, hypoxia or H_2_O_2_ conditions with or without pretreatment of 1 mM NAC or 1 μM wortmannin for 30 min. **a** Total cell lysates were immunoblotted with antibodies to acetyl α-tubulin^K40^, stathmin1 and P85. **b** The mRNA level of stathmin1 was detected with RT-qPCR. **c** Western blot of acetyl α-tubulin^K40^, PI3K and P85 (loading control) of HUVEC cells treated with PI3K siRNAs. **d** HUVEC cells were treated with hypoxia for 8 h with or without protein synthesis inhibitor cycloheximide and the transcription inhibitor actinomycin D, total cell lysates were immunoblotted with antibodies to acetyl α-tubulin^K40^ and P85. Representative results from three independent experiments were depicted here. The relative intensity of band was normalized to P85. The data are presented as the mean ± SEM. *P < 0.05

Stathmin1 is involved in microtubular depolymerization, regulation of microtubular dynamics [21, 22], and is a marker of PI3K pathway activation [23]. Thus, we focused on Stathmin1 to determine whether ROS affects the function of microtubules by regulating the expression of Stathmin1. Acetylated tubulin is widely used as a marker of stable microtubular structures [24–26]. The stability of microtubules was detected by western blot using an antibody to acetyl α-tubulin^K40^. Consistent with Figs. 1 and 2, the hypoxia and H_2_O_2_ treatments significantly decreased the protein levels of acetyl α-tubulin^K40^, indicating microtubule disassembly, and upregulated Stathmin1 protein and mRNA levels, while treatment with NAC or wortmannin increased the acetyl α-tubulin protein levels^K40^ and downregulated Stathmin1 protein and mRNA levels (Fig. 3a, b). To test whether PI3K/Akt regulates microtubular stability, we treated HUVECs with PI3K short interfering RNAs (siRNAs) and detected the expression of acetyl α-tubulin^K40^. Two of the four PI3K siRNAs tested inhibited the protein levels of acetyl α-tubulin^K40^ (Fig. 3c). PI3K is widely known as a regulator of gene transcription [27]; thus, to determine whether PI3K regulates microtubular stability in a transcription-dependent manner, we detected the expression of acetyl α-tubulin^K40^ in the presence of the mRNA synthesis inhibitor actinomycin D and the protein synthesis inhibitor cycloheximide with or without wortmannin (Fig. 3d). Neither actinomycin D nor cycloheximide inhibited acetyl α-tubulin expression^K40^, whereas wortmannin inhibited microtubular stability in the presence of actinomycin D and cycloheximide.

### ROS decreases Stathmin1 expression and blocks the interaction between PI3K and Stathmin1

**Fig. 4:**
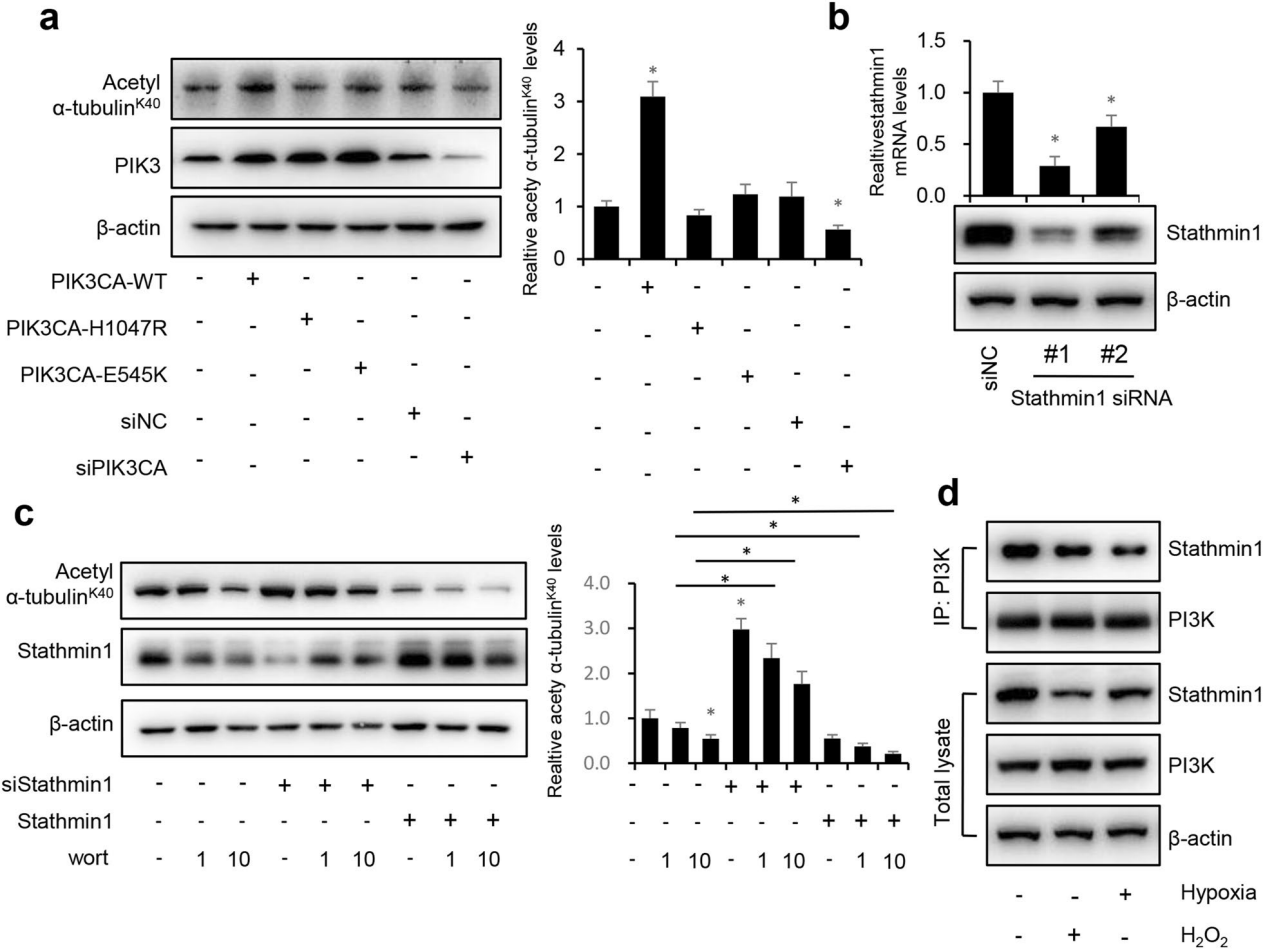
ROS decreases the expression of Stathmin1 and blocks the interaction between PI3K and Stathmin1. **a** Western blot of acetyl α-tubulin^K40^, PI3K and P85 (loading control) in hypoxia HUVEC cells expressing PIK3CA-WT, PIK3CA-H1047R and E545K or treated with PIK3CA siRNA. **b** Western blot of Stathmin1 siRNA-treated HUVEC cell using antibodies to Stathmin1 and β-actin (loading control). **c** HUVEC cells were transfected with Stathmin1 siRNA or Stathmin1 expression vector and treated with or without 1 μM or 10 μM wortmannin, total lysates were immunoblotted with antibodies to acetyl α-tubulin^K40^, Stathmin1 and P85. **d** Immunoprecipitation of PI3K from normoxia, hypoxia and H_2_O_2_ treated HUVEC cells using endogenous protein and immunoblotted for Stathmin1 and PI3K. Representative results from three independent experiments were depicted here. The relative intensity of band was normalized to P85. The data are presented as the mean ± SEM. *P < 0.05

To examine the regulation of PI3K in microtubular stability, we transfected expression vectors that expressed wild-type constitutively active (PIK3CA-WT) and kinase-dead (PIK3CA-H1047R and E545K) mutants then knocked down endogenous PI3K with siRNA. PIK3CA increased the expression of acetyl α-tubulin^K40^, suggesting that this active form of PI3K regulates microtubular stability. PIK3CA-H1047R and E545K are the most commonly used mutations leading to amino acid changes in the kinase domain, and cannot be tyrosine phosphorylated. These two mutations did not rescue the expression of acetyl α-tubulin K40. Knockdown of PIK3CA induced downregulation of acetyl α-tubulin^K40^ (Fig. 4a). These results suggest that the active form of PI3K is the primary mediator of microtubular disassembly.

As microtubular stability is independent of PI3K transcriptional factor function, we examined whether PI3K regulates microtubular stability via Stathmin1, a PI3K activation marker and inhibitor of microtubule polymerization. Depleting Stathmin1 with siRNA should have a similar effect as wortmannin and therefore Stathmin1 siRNA should make wortmannin more effective at inducing microtubular disassembly. Stathmin1 knockdown with one of the two siRNAs increased expression of acetyl α-tubulin^K40^ and wortmannin was more effective for protecting microtubular stability when Stathmin1 was knocked down (Fig. 4b, c). Overexpressing Stathmin1 significantly decreased the expression of acetyl α-tubulin^K40^ and promoted the effects of wortmannin on microtubular disassembly. These results suggest that the PI3K/Stathmin1 pathway regulates microtubular stability. However, it is unknown how PI3K regulates Stathmin1 and the effects of hypoxia and ROS. Thus, a co-immunoprecipitation assay was performed (Fig. 4d). As results, PI3K directly interacted with Stathmin1, and the hypoxia and H_2_O_2_ treatments blocked their interaction.

## Discussion

Cardiovascular disease remains the leading cause of mortality in Europe, causing almost 4.1 million deaths per year, or 46% of all deaths [28]. Impairment in local blood flow changes the microenvironment leading to hypoxia, which is a common pathological course [29]. ECs control many physiological and pathological actions, including inflammatory cell recruitment, regulation of vascular resistance and initiation of coagulation. Thus, malfunctioning ECs initiate many cardiac vascular diseases. Microtubular dynamics have a close relationship with proliferation, survival, migration, and the barrier integrity of ECs, which play an important role repairing the vasculature to prevent vasculopathy [30]. However, the functional role of hypoxia-induced oxidative stress in the balance of the EC microenvironment and the molecular mechanisms involved remain undefined.

In this study, we utilized HUVECs as a model to explore the structural and functional changes in microtubules and the mechanism of how hypoxia affects EC function. The results showed that hypoxia induced disassembly and depolymerization of microtubules, which can be mimicked by colchicine and prevented by paclitaxel. Paclitaxel protected HUVEC functions, whereas colchicine mimicked the hypoxic damage, suggesting that hypoxia induces EC malfunction by destroying the microtubular structure.

Hypoxia usually induces oxidative stress by promoting ROS production in ECs [31]. As shown in Fig. 2, the ROS level in HUVECs increased significantly under the hypoxic condition. ROS are upstream of the PI3K/Akt pathway [32] and Stathmin1 is associated with PI3K activity [22, 23, 33]. Thus, we determined whether the ROS/PI3K/Akt pathway regulates HUVEC function and microtubular structure. NAC and wortmannin were added to hypoxia-treated HUVECs and either blocked ROS or inhibited the PI3K/Akt pathway. Both reversed EC function and ameliorated the damaged microtubular structure, suggesting that ROS may be the intermediate between hypoxia and microtubular destruction, and hypoxia induced a malfunction in ECs through ROS production and activation of the PI3K/Akt pathway.

Hypoxia and H_2_O_2_ treatment significantly decreased the expression of acetyl α-tubulin^K40^ and increased Stathmin1 protein and mRNA levels. NAC and wortmannin recovered the level of acetyl α-tubulin^K40^ and inhibited expression of Stathmin1, indicating that hypoxia and ROS damaged the microtubular structure in a Stathmin1-dependent manner. PI3K knockdown destroyed the microtubular structure but not via transcription (Fig. 3c, d). PI3K is an essential factor in hypoxia-induced apoptosis [34], angiogenesis [35], and inflammation [36]. Here, we demonstrated that the microtubular structure is a PI3K-dependent function, revealing a new mechanism of the PI3K pathway in HUVECs. PIK3CA, the catalytic subunit of class IA PI3K, plays a major role in PI3K-related progression [37]. PIK3CA-H1047R and E545K cannot be tyrosine phosphorylated and are primarily monomeric [19]. We discovered that PIK3CA-WT increased microtubular stability, but PIK3CA-H1047R and E545K had no similar effect. PIK3CA knockdown further decreased microtubular stability. Overall, these results suggest that the active form of PI3K is the primary mediator of microtubular stability. Knockdown of Stathmin1 improved, whereas overexpression of Stathmin1 inhibited microtubular stability. Wortmannin damaged microtubular stability in a dose-dependent manner. Hypoxia and ROS blocked the interaction between transcription factor PI3K and Stathmin1.

Hypoxia, ROS, and disassembly of microtubules impair the proliferation, survival, permeability, and functions of ECs. However, in this study, we demonstrated that oxidative stress resulting from activation of cellular ROS production is a pathological course of hypoxia, which further disrupted microtubular dynamics and EC functions. We also demonstrated that the PI3K/Stathmin1 pathway plays an important role in the hypoxia-destroyed microtubular stability. As hypoxic-injured EC cells are frequently observed in cardiovascular disease and diabetes, it would be interesting to examine whether patients with cardiovascular disease and diabetes respond better to PI3K inhibitors or a microtubular stabilizer in clinical trials.

## Conclusions

Our results demonstrate that hypoxia disturbed the ultrastructure and polymerization of microtubules and induced dysfunction of ECs. Furthermore, ROS activated the PI3K/Akt pathway and downregulated the expression of Stathmin1 by blocking the interaction between PI3K and Stathmin1, which renders PI3K inhibitors and microtubule stabilizers as promising therapeutic drugs in hypoxia-induced vascular disease.

## Abbreviations

EC: endothelial cells
HUVEC: human umbilical vein endothelial cells
ROS: reactive oxygen species
TEM: transmission electron microscope
PI3K: phosphatidylinositol-3 kinases
DAPI: 4′,6-diamidino-2-phenylindole, dihydrochloride
HIF: hypoxia-inducible factor 1
FBS: fetal bovine serum
TNF: tumour-necrosis factors
TGF: transforming growth factor
NAC: *N*-acetyl cysteine
